# Varicella-zoster virus infections in immunocompromised patients - a single centre 6-years analysis

**DOI:** 10.1186/1471-2431-11-31

**Published:** 2011-05-10

**Authors:** Verena Wiegering, Judith Schick, Meinrad Beer, Benedikt Weissbrich, Stefan Gattenlöhner, Hermann J Girschick, Johannes Liese, Paul G Schlegel, Matthias Eyrich

**Affiliations:** 1 Dept. of Pediatric Hematology-Oncology, Pediatric Stem Cell Transplantation Program, University Children's Hospital Wuerzburg, Germany; 2 Dept. of Pediatric Infectious Diseases, Immunology and Rheumatology, University Children's Hospital Wuerzburg, Germany; 3 Dept. of Pediatric Radiology, University of Wuerzburg, Germany; 4 Institute for Virology and Immunbiology, University of Wuerzburg, Germany; 5 Dept. of Pathology, University of Wuerzburg, Germany

**Keywords:** varicella-zoster virus, immunosuppression, pediatrics, cidofovir

## Abstract

**Background:**

Infection with varicella-zoster virus (VZV) contemporaneously with malignant disease or immunosuppression represents a particular challenge and requires individualized decisions and treatment. Although the increasing use of varicella-vaccines in the general population and rapid initiation of VZV-immunoglobulins and acyclovir in case of exposure has been beneficial for some patients, immunocompromised individuals are still at risk for unfavourable courses.

**Methods:**

In this single center, 6-year analysis we review incidence, hospitalization and complication rates of VZV-infections in our center and compare them to published data. Furthermore, we report three instructive cases.

**Results:**

Hospitalization rate of referred children with VZV-infections was 45%, among these 17% with malignancies and 9% under immunosuppressive therapy. Rate of complications was not elevated in these two high-risk cohorts, but one ALL-patient died due to VZV-related complications. We report one 4-year old boy with initial diagnosis of acute lymphoblastic leukemia who showed a rapidly fatal outcome of his simultaneous varicella-infection, one 1.8-year old boy with an identical situation but a mild course of his disease, and an 8.5-year old boy with a steroid-dependent nephrotic syndrome. This boy developed severe hepatic involvement during his varicella-infection but responded to immediate withdrawl of steroids and administration of acyclovir plus single-dose cidofovir after nonresponse to acyclovir after 48 h.

**Conclusion:**

Our data show that patients with malignant diseases or immunosuppressive therapy should be hospitalized and treated immediately with antiviral agents. Despite these measures the course of VZV-infections can be highly variable in these patients. We discuss aids to individual decision-making for these difficult situations.

## Background

Infections with varicella-zoster virus (VZV) are usually considered benign infections. However, severe complications including bacterial superinfections, coagulopathies, and central nervous system manifestations with a potentially fatal or long term disabling outcome can occur [1,2]. The classical clinical presentation is characterized by mucocutaneous involvement and a low mortality rate in immunocompetent children. In contrast, primary varicella-infections are potentially life-threatening in immunocompromised patients, especially in those whose immune system has been suppressed by diseases such as acute lymphoblastic leukaemia (ALL) or by multiagent chemotherapy treatment including corticosteroids.

After introduction of antiviral treatment with acyclovir and varicella-zoster immune globulin (VZIG), mortality rate of varicella-infections in children with immune suppression has decreased significantly (<1%). Before introduction of antiviral therapy, the mortality rate of varicella infections in children with cancer was reported to be 7%, with numbers reaching up to 55% in cases with visceral involvement [3-6]. In immunocompromised patients the diagnosis of varicella may be obscured by atypical or even absent skin lesions in combination with systemic viral disease [5,7,8].

The management of VZV-infection in immunocompromised patients can represent a challenging dilemma for the treating physician due to the balance between infection and underlying disease. The limited published evidence to support specific therapeutic management processes prompted us to report our recent experience in clinical decision making in patients with overt VZV-infections under immunosuppression and/or malignancies.

## Methods

All patients with VZV-infections treated in the emergency room of the University Children's Hospital Würzburg between January 2004 and March 2010 were identified using ICD-10 hospital discharge diagnosis system and their medical charts were reviewed for hospitalization, treatment, treatment duration, occurrence of complications, and outcome. The identified patient cohort was further divided into subgroups with either a underlying malignancy, disorders requiring immunosuppressive therapy or none of these two conditions. Through this we could calculate local hospitalization, treatment, and complication rates of VZV infections. Furthermore, rate and severity of VZV-related complications were compared between healthy and immunosuppressed children. Acquired data were compared with a review of the literature. Keywords for literature search were varicella zoster virus infection, immunosuppression, malignancy and complications. Finally, three instructive cases of children with VZV-infections and high-risk conditions are reported in detail.

Patients and/or guardians treated in our academic hospital gave informed consent to scientific analysis and anonymized publication of their medical data in accordance with the Declaration of Helsinki. The ethical committee of the University Hospital Würzburg has determined that for analysis and publication of single center case series this informed consent is sufficient and no specific review of retrospective data analysis projects are required.

## Results

A total of 119 children with VZV-infections have been seen at the emergency room of the University Children's Hospital Würzburg between January 01, 2004 and March 31, 2010. The median age of these 119 children was 3.8 years (range: four weeks to 18 years). 54 out of these 119 patients (45%) were hospitalized. Out of the 54 admitted children 40 (74%) did not have a severe underlying disease, 9 patients (16.7%) had an oncologic disorder and 5 patients (9.3%) received an immunosuppressive treatment. VZV-infection and/or risk factors for a complicated course were the prime indication for in-patient care in all cases. None of the patients with malignancies or immunosuppression were treated on an outpatient basis and all received intravenous acyclovir as antiviral treatment immediately. From the 40 patients without a severe underlying disease 6 (15%) developed complications of their VZV-infection (5 cerebellitis, 1 coagulopathy). In each of the high-risk groups one patient developed a complicated course (Table 1), resulting in a complication rate of 11% versus 20% (malignant disease vs. immunosuppression, respectively).

**Table 1:** Patient characteristics

patient	gender	age [years]	start VZV exanthema until hospital admission	organ involvement	start antiviral therapy	underlying disease/time point of therapy	duration of antiviral therapy	outcome
Oncologic patients								
1	m	4	1 day	skin, peripheral blood, bronchial secret, pleural effusion, ascites, myocard, lung, liver, kidneys	immediately, acyclovir i.v.	T-ALL, diagnosis	until death (2^nd ^Day)	death of VZV
2	m	6	same day	skin	immediately, acyclovir i.v.	c-ALL, maintenance therapy	7 days	alive, no sequelae
3	m	4	same day	skin	immediately, acyclovir i.v.	neuroblastoma, maintenance with retinoic acid	7 days	alive, no sequelae
4	m	5	same day	skin	immediately, acyclovir	T-ALL, consolidation chemotherapy, herpes zoster	7 days	alive, no sequelae
5	m	8	same day	skin	immediately, acyclovir i.v.	T-ALL, induction chemotherapy	5 days	alive, no sequelae
6	f	1	same day	skin	immediately, acyclovir i.v.	astrocytoma, induction therapy	7 days	alive, no sequelae
7	m	7	same day	skin	immediately, acyclovir i.v.	pro-B-ALL, allogeneic BMT, d +114, herpes zoster	7 days	DOD
8	m	7	same day	skin	immediately, acyclovir i.v.	pro-B-ALL, allogeneic BMT, d +60, herpes zoster	7 days	DOD
9	m	1.8	3 days later	skin	immediately, acyclovir i.v.	cALL, diagnosis	49 days	alive, no sequelae
Immunocompromised patients								

10	m	8	1 day	skin, liver	immediately, acyclovir and cidofovir	nephrotic syndrome	7 days	alive, no sequelae
11	f	7	1 day	skin	immediately, acyclovir i.v.	juvenile idiopathic arthritis (MTX, prednisone)	3 days i.v., 5 days p.o.	alive, no sequelae
12	f	5	same day	skin	immediately, acyclovir i.v.	juvenile idiopathic arthritis (MTX, prednisone)	3 days*	alive, no sequelae
13	f	4 weeks	3 days	skin	immediately, acyclovir i.v.	neonatal	4 days	alive, no sequelae
14	m	3	same day	skin	immediately, acyclovir i.v.	pemphygoid, enteropathy (prednisone)	14 days	alive, no sequelae

All patients were admitted due to VZV-infections and/or the presence of risk factors for a complicated course of the disease.

*Antiviral therapy was stopped after only three days due to acute, but transient renal failure under acyclovir.

Abbreviations: BMT = bone marrow transplantation, c-ALL = common acute lymphoblastic leukemia, d = day, i.v.= intravenously, MTX = methotrexate, DOD = dead of underlying oncologic disease, p.o.= orally, pro-B-ALL = B-progenitor acute lymphoblastic leukemia, VZV = varicella zoster virus

Among the 9 children with underlying oncologic diseases 7 were diagnosed with acute lymphoblastic leukaemia (ALL), 1 with neuroblastoma and 1 with an astrocytoma of the CNS. Children with immunosuppressive therapy were treated due to nephrotic syndrome (n = 1, patient 10), pemphygoid and enteropathy (n = 1) and rheumatic disease (n = 2). We recorded one VZV-related death in a child with ALL (patient 1).

## Case presentations

### Patient 1

A 4 year old boy presented with a two day history of macro-hematuria, fatigue, and emesis as well as a one day history of varicella-like skin lesions. An exposure to varicella three to four weeks ago had been reported. Occurrence of blue spots (hematoma) had been noted on his lower legs in the last few days before admission. There was no history of epistaxis or other signs of bleeding, respectively.

**Figure 1:**
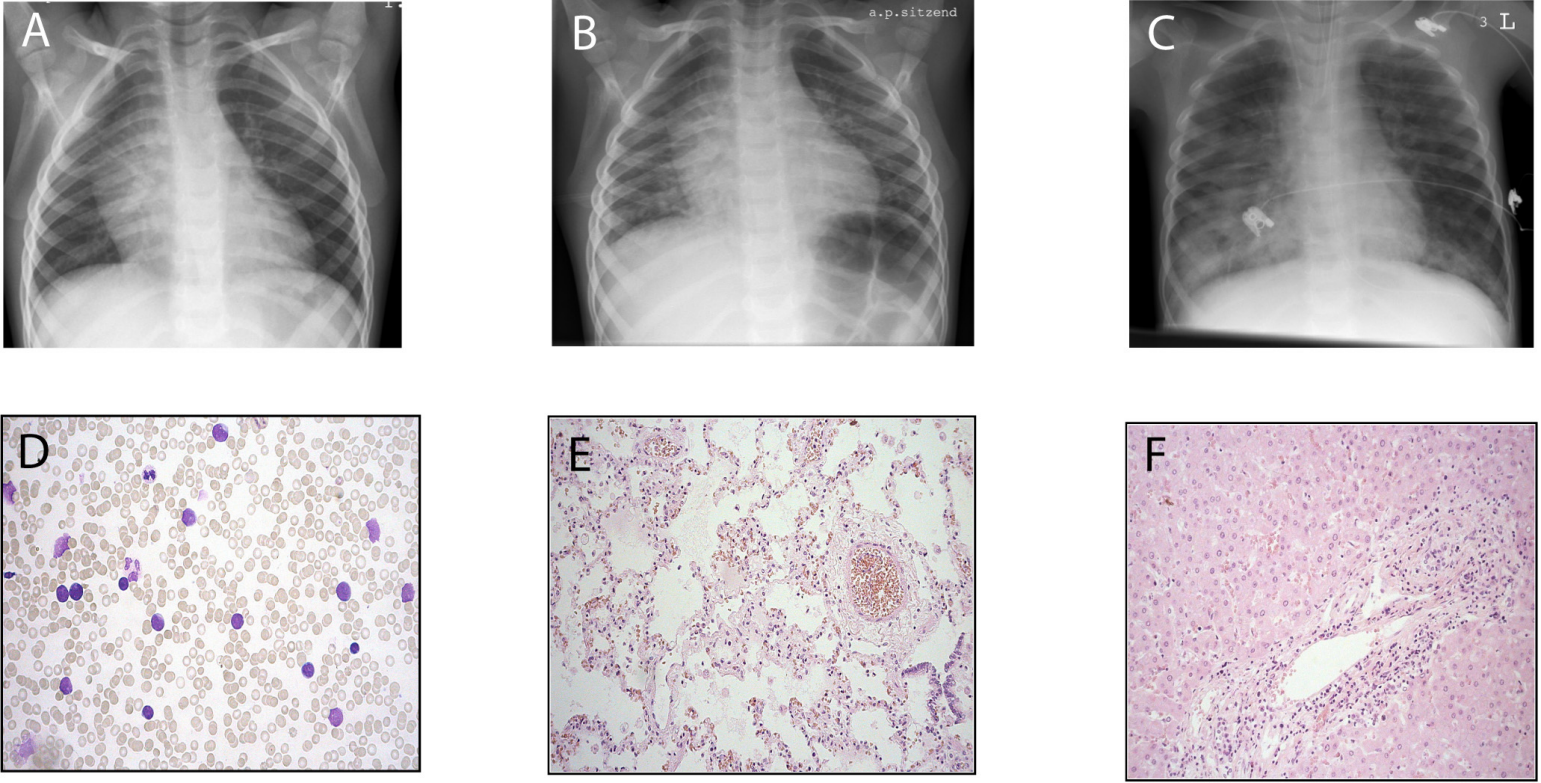
**Serial chest X-rays displaying the fulminant course of disease**. Upon admission a mediastinal tumour was described (A). 23 hours after admission, the mediastinal tumour diminished, however, bilateral pulmonary infiltrates could be noted (B) which progressed rapidly (C, 6 hours later). D) bone marrow aspiration at diagnosis: 80% T-lymphatic blasts, E) histology of lung parenchyma: pulmonary capillaries obstructed by lymphatic blasts, no interstitial infiltrations of the lung parenchyma, pulmonary edema due to leukostasis (autopsy tissue), F) Histology of liver: liver cells with inflammatory signs (autopsy tissue).

Immediate blood and bone marrow diagnostic tests confirmed the initial suspicion of the simultaneous clinical manifestation of acute varicella-infection and acute lymphoblastic leukaemia. Leukocytosis (leukocytes 69,810/μl) accompanied with mild anemia (Hb 11.5 g/dl) and thrombocytopenia (62,000/μl), an enlargement of the thymus in conventional chest X-ray (Figure 1A) and 80% blast cells in bone marrow revealed an acute lymphoblastic leukaemia (Figure 1D), which exhibited a T-precursor phenotype in subsequent flow cytometric assessment. Varicella-infection was proven by VZV-DNA detection in vesicles and blood, while both VZV-IgM and -IgG were negative at admission (48 hours after the first appearance of skin lesions).

The patient was clinically in a stable cardiopulmonary condition, so we refrained from starting antileukemic therapy with corticosteroids to allow for viral control. Instead, immediate treatment with intravenous acyclovir (45 mg/kg BW/d) and specific immunoglobulin (Varitect 100 mg/kg BW) was initiated.

In the first few hours thereafter the patient remained in stable clinical condition. Due to leukocytosis and the inherent risk of tumor-lysis-syndrome he received hydration plus diuretic therapy, resulting in an even fluid balance. Even without receiving corticosteroids a progressive reduction of leukocyte counts from 70,000/μl initially to 9,000/μl was observed, in addition the thymus decreased in size.

Four hours after initiation of treatment, a minor oxygen demand could be noted, the current chest x-ray showed increasing pulmonary infiltrates (Figure 1B). Otherwise, the clinical condition remained stable, the patient was able to eat and play.

During the next few hours the previously stable clinical condition worsened dramatically with increasing oxygen demand, but despite maximal oxygen supply the patient showed signs of respiratory distress with a venous oxygen saturation of below 90%.

Subsequently, the patient was transferred to the intensive care unit (ICU), where mechanical ventilation became necessary. The patient subsequently developed a severe hemorrhagic pulmonary edema as evidenced by bloody-foamy tracheal secretion (Figure 1C). At this time point the patient showed centralisation, disseminated intravascular coagulation, and fulminant multi-organ failure to which he succumbed only three hours after the admission to the ICU.

Post mortem autopsy report showed an enlargement and consolidation of thymus, spleen, and lymph nodes, pulmonary edema with subpleural echymosis. Furthermore, a dilatation of the right ventricle, leukostasis in the lungs (Figure 1E), liver (Figure 1F) and spleen, shock-kidneys, signs of disseminated intravascular coagulopathy in several organs together with peritoneal, pericardial and pleural effusions could be observed. VZV-DNA could be detected in peripheral blood, bronchial lavage, pleural effusion, ascites, myocard, lung, liver and kidneys. Pericardial effusion was negative for VZV.

Endogenous cortisol production was measured retrospectively in backup serum samples. There was a clear increase in endogenous cortisol production from 396 ng/ml at admission to values beyond the detection limit (>500 ng/ml) during the course on the ICU [age adapted normal values 2.5-230 ng/ml at 8 a.m.].

### Patient 9

A 20 month old boy was admitted to our hospital with signs of anemia and thrombocytopenia. Subsequent evaluation revealed acute lymphobastic leukemia of preB-precursor phenotype (cALL). Leukocytes in peripheral blood were 14,880/μl (76% blasts), Hb 6.1 g/dl, thrombocytes 8,000/μl, and 95% blast cells in bone marrow. Careful evaluation of medical history revealed that the patient's sister has been diagnosed with chickenpox two weeks ago. The patient had received a single dose of varicella-vaccination 7 months earlier, but was negative for VZV-IgG and -IgM at presentation. Due to the stable condition of the patient and this significant risk factor we postponed antileukemic therapy with steroids and only initiated antiviral treatment (acyclovir 45 mg/kg BW/d), hydration and close clinical surveillance of the patient. In fact, 3 days after admission the boy developed skin lesions (approximately 20-30 lesions) compatible with primary varicella-infection. However, these lesions were described as "atypical varicella" by a consulting dermatologist due to the reduced inflammatory halo around the vesicles. The content of the vesicles proved to be positive for VZV-DNA, while repeated PCR-testing in blood did not give positive results. Under continuous hydration, antiviral therapy with acyclovir and a wait-and-see strategy the lesions slowly disappeared without further complications within two weeks. A DNA-swab from a vesicle 10 days after the appearance of the first one was negative for VZV. Three days later (16 days after admission to the hospital) therapy with corticosteroids as the first part of ALL-induction therapy was initiated. The boy showed a fast and sustained prednisone-response of his ALL and did not reactivate VZV during subsequent chemotherapy. Acyclovir medication was continued until the end of the induction protocol. The patient did not developed anti-VZV antibodies during ALL treatment.

### Patient 10

**Figure 2:**
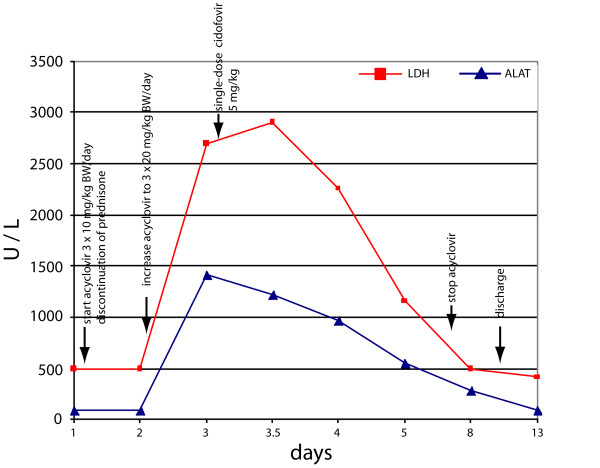
**Timely relationship between laboratory values (LDH = lactate dehydrogenase and ALAT = alanine aminotransferase) and therapeutic measures in patient 10**. 48 h after discontinuation of steroid treatment and initiation of antiviral treatment with acyclovir, liver enzymes continued to increase whereas liver function parameters declined (not shown). Addition of cidofovir (single dose) to acyclovir was followed by a rapid and sustained normalization of liver function parameters.

A 8.5 year old boy with a chronic nephrotic syndrome presented with typical varicella skin lesions on his trunk and pilous head, leg pain, and fatigue. Varicella-infection could be confirmed by VZV-DNA detection in vesicles, while both VZV-IgM and -IgG were still negative at this early time point of infection. Due to his underlying disease the patient had received prednisolone 60 mg/m^2 ^for the last month. Therefore an immediate intravenous therapy with acyclovir (30 mg/kg BW/d) was started; prednisolone was discontinued. Despite these measures the patient's clinical condition worsened with decreasing liver function tests (maximum of laboratory findings on the third day after admission [age adapted reference range]: GOT: 1012 U/l [12-51], GPT: 1409 U/l [7-44], GGT: 29.3 U/l [5-25], LDH: 2697 U/l [88-298], Albumin 2.8 g/dl [3.5-5.5] and Cholinesterase: 4000 U/L [5500-13000], see Figure 2) and signs of disseminated intravascular coagulation (D-dimers 20.98 mg/l [normal range 0-0.5]). However, vital parameters remained stable during the hospital stay. Because of the rapid clinical deterioration under acyclovir therapy we decided to administer a single dose of cidofovir (5 mg/kg) as an experimental therapy due to the critical illness of the patient after obtaining informed parental consent. The decision for cidofovir was based on the conception that a second antiviral drug with a different mode of action (acyclic nucleoside phosphonate without the need for activation by the viral thymidine kinase) [9] and a proven effect on many herpesviridae [10] could favorably influence the course of the disease. In the following days, clinical condition and laboratory findings improved and the boy could be discharged nine days later. No renal side-effects of cidofovir treatment could be observed.

## Discussion

Immunocompromised patients are threatened by severe infections, in part by infectious agents which rarely cause complications in non-immunosuppressed children. VZV-infections in this patient cohort have an increased risk of serious morbidity and even death [1]. Before the era of antiviral therapy, disseminated and/or fatal VZV infections have been described in children with cancer under chemotherapy, especially in acute lymphoblastic leukemia [3]. Recently, an increasing general awareness of varicella-related complications has led to the assumption that hospitalization with subsequent severe complications occurs in approximately 20% of VZV-infected, immunocompromised patients [1]. Most frequently described complications of VZV-infection during chemotherapy and immunosuppression are interstitial or necrotising pneumonia, viral hepatitis with acute liver failure, coagulopathies or bacterial superinfections [8,11]. In contrast, VZV-infected immunocompetent children were reported to have a higher incidence of neurologic complications (32% vs. 15%, immunocompetent vs. immunosuppressed children, respectively) or cutaneous bacterial superinfections (30% vs. 7%) [1,2]. Interestingly, infections manifesting as herpes zoster in these immunocompromised patients were not associated with more complications with exception of a generalised zoster rash, which has been reported to occur in 25% [12]. Our three patients with herpes zoster manifestations (patient 4, 7, and 8) did not develop any complications.

Out of 119 patients seen with VZV infections we could identify 14 immunocompromised/oncologic patients in six years (11.8%). All were immediately hospitalized for therapeutic antiviral treatment. With the exception of one case (patient 1), early and consequent antiviral treatment was effective in preventing severe courses so that the overall complication rate in the two high-risk groups was not elevated compared to the otherwise healthy group. This is in contrast to other reports in the literature, which show a higher risk of complications and death in leukemic children with VZV infections [1,3]. However, the only death occurred in a child with ALL, a disease that was overrepresented compared to other malignancies (7 patients with ALL vs. 1 case with neuroblastoma and 1 case with an astrocytoma).

Several reports describe liver failure without other organ manifestations due to VZV in immunocompromised patients [5,7,8,13], which seems to be a typical clinical manifestation in oncology patients. Rapid deterioration of liver function in patient 10 under acyclovir therapy suggested such a course with a presumed nonresponse to this drug. Thus, we expanded antiviral therapy by the application of a single dose of cidofovir. This decision was based on the relatively beneficial toxicity profile of cidofovir after short term application [14], *in-vitro *data showing antiviral activity against acyclovir- and foscarnet-resistant VZV-strains [9], experience with the efficiency of cidofovir in herpesviridae infections in oncology patients [10], and the long half-life after a single dose. One day after the application of cidofovir, liver parameters improved and returned to normal within one week. However, it remains unclear, whether this success is attributable to cidofovir or rather to the improving immune function after cessation of steroids two days earlier. Anecdotal reports exist which demonstrate good responses of acyclovir-resistant VZV-strains to alternative substances such as forscarnet or cidofovir [12,15]. However, an acyclovir-resistant strain can hardly be claimed in this situation, as these are mainly isolated from immunocompromised patients after long-term acyclovir administration. The efficacy of alternative viral drugs such as foscarnet or cidofovir alone or in combination with standard antiviral substances especially in high-risk situations certainly warrants further investigation in larger, controlled trials.

Our two cases of VZV-infections in children with newly diagnosed ALL exhibited highly divergent courses. Whereas in patient 9, ALL therapy could be postponed safely until viral control was obtained, patient 1 took a fulminant fatal course within hours after admission despite all therapeutic interventions. Although we could not detect protective anti-VZV-titers, it can be discussed whether patient 9 may have benefitted from the previous single varicella-vaccination seven months earlier. The autopsy report in patient 1 described *pulmonary leukostasis *as the most likely explanation for the fatal respiratory failure and right ventricle dilatation. Several adhesion molecules were upregulated on pulmonary endothelial cells (data not shown). We hypothesize that in this case pulmonary VZV-infection triggered a preferential clustering of T-ALL blasts in pulmonary capillaries via an increased expression of adhesion molecules. Interestingly, whereas VZV-DNA could be found in nearly every organ, blast cells could only be found in terminal capillary vessels. This phenomenon has already been described for myeloid blasts [16]. Together with the increased endogenous cortisol production, this preferential sequestration of blasts into the pulmonary circulation provides an explanation for both the rapidly diminishing leukocyte counts and for the progressive right heart and respiratory failure. It remains speculative whether an initiation of steroid treatment despite systemic VZV-infection could have prevented obstruction of pulmonary circulation system.

Routine vaccination against varicella, as recently established in Germany, has a great potential to significantly reduce the burden of this disease, if sufficiently high coverage is reached in the target population. Vaccination can not only reduce the number of infections and infection-related complications but also provide herd immunity. Especially immunocompromised children may profit from this. In Germany it could already be demonstrated, that complications and mortality of varicella infections significantly decreased since the introduction of varicella-vaccines in the standard childhood vaccination schedule [17].

## Conclusions

Our retrospective, 6-year single centre data show that immunocompromised children with VZV-infections are frequently hospitalized and rapidly receive antiviral therapy. Using this approach even in this high-risk patient cohort severe VZV-related complications are rare, however, they cannot be prevented in all cases, especially in newly diagnosed immunocompromised children. Parameters that may predict an adverse clinical outcome can be duration and intensity of immunosuppression, biological course and phenotype of leukemia (T-versus precursor B-cell-phenotype), time post transplantation/chemotherapy and degree of immune reconstitution achieved so far. Close monitoring of organ function and viral load can identify patients not responding to acyclovir who may then be considered candidates for alternative therapies such as foscarnet or cidofovir. However, this approach should be evaluated in larger patient series. Additional experimental data will have to show, if a relationship between T-phenotype ALL, systemic inflammation and a preferential homing of blasts into the pulmonary circulation system can be established.

## List of abbreviations used

BMT: bone marrow transplantation; c-ALL: common acute lymphoblastic leukaemia; DNA: deoxyribonucleic acid; ICU: intensive care unit; Ig: immunoglobulin; PCR: polymerase chain reaction; VZV: varicella zoster virus; VZIG: varicella zoster immune globulin;

## Competing interests

The authors declare that they have no competing interests.

## Authors' contributions

VW chart review, data analysis, manuscript preparation, JS preparation of radiologic images, data analysis and preparation of the manuscript, MB realization and interpretation of radiologic images, BW summary of virology data, manuscript revision, SG provided the pathologic images, HJG provided medical care, literature search, manuscript preparation, JL literature search, manuscript revision, PGS provided medical care, literature search, manuscript revision, ME provided medical care, data analysis, manuscript preparation, literature search.

All authors: final review of the manuscript.

## Pre-publication history

The pre-publication history for this paper can be accessed here:

http://www.biomedcentral.com/1471-2431/11/31/prepub
